# Spatio-Temporal Graph Autoencoder for Sensor Data Reconstruction in Vineyard Microclimate Monitoring

**DOI:** 10.3390/s26144368

**Published:** 2026-07-09

**Authors:** Filippo Costanti, Irene Cappelli, Monica Bianchini, Ada Fort

**Affiliations:** Department of Information Engineering and Mathematics, University of Siena, 53100 Siena, Italy; irene.cappelli2@unisi.it (I.C.); monica.bianchini@unisi.it (M.B.); ada.fort@unisi.it (A.F.)

**Keywords:** denoising autoencoder, environmental monitoring, gated recurrent unit with decay, graph sample and aggregate, internet of things, time series reconstruction

## Abstract

Continuous monitoring of climatic variables is essential for precision viticulture and data-driven decision support systems. However, agricultural sensor networks are frequently affected by missing data due to hardware failures, communication issues, or maintenance interruptions. In this work, we propose a spatio-temporal graph-based autoencoder for reconstructing missing temperature and relative humidity time series collected from a five-node vineyard sensor network over a two-year period. The model combines a GRU-based temporal encoder, augmented with a time-decay imputation mechanism applied to the input data, with a GraphSAGE spatial module, enabling the joint exploitation of temporal dynamics and inter-node spatial correlations. Experimental results on real-world data show that the proposed approach achieves accurate reconstruction under controlled missing-data scenarios generated through structured artificial masking. For moderate corruption levels (p=0.3), the model attains reconstruction losses of 0.003 for temperature and 0.005 for humidity using short temporal windows (*L* = 36~3 h), corresponding to MAE values below 0.03 °C and 0.1%, respectively. Even at higher corruption levels (p=0.7), performance remains stable, with losses below 0.008 and 0.011, and MAE values within 0.05 °C and 0.17%. The results highlight a trade-off between temporal context and reconstruction accuracy: shorter windows yield lower absolute errors under moderate corruption whereas, under extreme data loss (p=0.9), the longer windows (*L* = 144~12 h) reduce the composite temperature reconstruction loss from 0.027 to 0.021. Additionally, temperature is consistently reconstructed more accurately than humidity, reflecting its smoother dynamics and stronger spatial coherence.

## 1. Introduction

Precision viticulture has progressively transitioned from empirical management practices to data-driven decision frameworks supported by distributed sensing infrastructures [1]. Within vineyard ecosystems, climatic variables such as air temperature, relative humidity (RH), solar radiation, and precipitation directly influence plant physiology, evapotranspiration rates, pathogen development, and fruit quality [2]. Since these processes are highly sensitive to local environmental fluctuations, continuous and spatially distributed monitoring is essential to capture the fine-scale variability that characterizes vineyard terrains.

Wireless sensor networks (WSNs) based on low-power internet of things (IoT) technologies have become fundamental for smart agriculture systems [3]. By enabling long-term deployment of sensing nodes across heterogeneous plots, these platforms generate high-resolution time series that can feed predictive models for irrigation scheduling, disease risk estimation, and yield optimization. In this context, the value of the sensing infrastructure is not limited to data acquisition alone; rather, it lies in its integration within a complete digital pipeline that includes storage, preprocessing, modeling, and decision support.

However, real-world IoT deployments are inherently exposed to reliability constraints. Battery-powered nodes operating under outdoor conditions may experience energy instabilities, sensor degradation, or hardware faults. At the communication layer, low-power wide-area network (LPWAN) protocols such as long range wide-area network (LoRaWAN) introduce duty-cycle limitations and latency variability. These phenomena may result in irregular sampling, missing observations, and node-specific anomalies in the collected datasets. In terms of IoT reliability, these aspects should be regarded as inherent characteristics of distributed, resource-constrained systems rather than exceptional events [4].

Although these issues may appear primarily infrastructural, originating at the sensing and communication layers, they have direct implications for data-driven modeling. Both classical statistical models and modern machine learning (ML) approaches are sensitive to the temporal structure of the input data. Irregular sampling and missing observations, if not explicitly modeled, can alter the effective dependency structure of the series, leading to biased inference, degraded forecast accuracy, or reduced training stability. These effects are particularly relevant when temporal alignment across multiple sensing nodes is required, as in multi-node vineyard deployments. Indeed, inconsistent timestamps or heterogeneous data gaps across nodes can hinder comparability and the detection of physically meaningful microclimatic differences. Consequently, inadequate handling of missing or irregular data does not merely reduce the dataset size, it alters the statistical and dynamical properties of the signals, with direct effects on the prediction accuracy [5].

Sensor data reconstruction therefore represents a critical interface between IoT system reliability and ML robustness. Its role extends beyond simple gap filling: it ensures that the statistical properties of the reconstructed signals remain coherent with the physical dynamics of the vineyard microclimate [6]. Temperature and relative humidity, for instance, exhibit smooth diurnal cycles governed by radiative forcing and atmospheric mixing processes. Reconstruction strategies must preserve these dynamics without introducing artificial oscillations or attenuating variability. From a modeling perspective, reconstruction directly influences feature quality, temporal dependencies, and spatial comparability across nodes. Inadequate handling of missing data may lead deep learning models to learn artifacts rather than true environmental patterns. Conversely, physically informed interpolation and consistent temporal alignment can enhance the stability of training procedures and improve predictive accuracy.

In this work, we address the reconstruction of missing data using real-field air temperature and RH measurements collected from a five-node vineyard sensor network deployed under field conditions over a two-year period [7]. To jointly exploit temporal dynamics and spatial correlations among neighboring sensors, we propose a spatio-temporal graph-based autoencoder. The model combines a Gated Recurrent Unit (GRU) module to capture temporal dependencies under irregular sampling and missing observations, with a GraphSAGE (Graph Sample and Aggregate)-based spatial encoder operating on the graph representation of the sensor network. The experimental results support the view that, in distributed agricultural monitoring systems, reliable data reconstruction benefits from explicitly integrating network topology and environmental dynamics within a unified learning framework.

The paper is organized as follows. Section 2 reviews related work on missing data reconstruction and spatio-temporal modeling in environmental sensor networks. Section 3 describes the vineyard sensing infrastructure, the dataset collection and preprocessing, and the proposed spatio-temporal graph autoencoder. Section 4 presents the experimental results while Section 5 discusses the obtained outcomes. Finally, Section 6 draws the conclusions and outlines directions for future research.

## 2. Related Works

Recent research on sensor network modeling has progressively moved toward structure-aware learning architectures that explicitly incorporate both spatial topology and temporal dynamics into deep neural models. In this context, spatio-temporal graph learning frameworks have emerged as an effective paradigm for representing dependencies among geographically distributed sensors and irregular temporal observations.

Spatio-Temporal Graph Neural Networks (STGNNs) extend classical time-series models by integrating graph structures that encode spatial relationships among nodes. Early architectures such as the Spatio-Temporal Graph Convolutional Network (STGCN) [8], the Diffusion Convolutional Recurrent Neural Network (DCRNN) [9], and Graph WaveNet [10] demonstrated that combining graph-based spatial modeling with temporal learning mechanisms significantly improves forecasting accuracy in networked time-series data. These models typically integrate graph convolutional operators with recurrent modules or temporal convolutional layers in order to jointly capture spatial correlations and temporal dependencies [11].

Compared with traditional sequence-based models, spatio-temporal embeddings provide a richer representation of environmental sensing systems. In many real-world monitoring scenarios, measurements collected at different locations are not independent but are governed by spatially correlated physical processes such as atmospheric circulation, hydrological flows, or soil moisture diffusion. By embedding sensor observations into a graph representation, STGNNs explicitly model these spatial interactions, allowing information to propagate across neighboring nodes and enabling the network to capture both local and global spatial patterns. This joint spatial–temporal representation is particularly advantageous for environmental forecasting tasks, where the evolution of a variable at a given location depends not only on its historical behavior but also on the conditions of nearby regions. As a result, STGNN-based models have demonstrated strong performance in a variety of environmental monitoring applications, including air quality forecasting (e.g., PM_2.5_ prediction) [12], soil moisture estimation [13,14], and hydrological forecasting systems [15,16].

In parallel to spatio-temporal graph models, deep learning models based on temporal convolutional architectures have also been explored for environmental data reconstruction in sensor network applications. In particular, WaveNet-inspired models leveraging dilated causal convolutions have been proposed to infer unmeasured environmental variables from correlated meteorological observations, effectively capturing non-linear dependencies and long-range temporal patterns in vineyard monitoring datasets [17]. These approaches demonstrate that predictive reconstruction of environmental quantities can be interpreted as an implicit missing data recovery task, highlighting the potential of data-driven sequence modeling techniques in real-world agricultural sensing systems.

More recently, the use of self-supervised learning techniques have been explored, to improve the robustness of spatio-temporal models when dealing with noisy or incomplete sensor data. In particular, masked autoencoder strategies have gained attention as a general framework for representation learning. Originally introduced for visual representation learning [18], masked autoencoders have subsequently been extended to spatio-temporal data by reconstructing partially masked observations in both spatial and temporal domains [19]. Within the graph learning domain, several works have proposed masked graph autoencoder architectures that combine graph message passing with self-supervised reconstruction tasks. For instance, the Spatio-Temporal Graph Masked Autoencoder (STGMAE) framework introduces a generative self-supervised paradigm in which both node features and graph structures are partially masked during training. The model learns to reconstruct the missing information, enabling more robust latent representations for noisy and sparse spatio-temporal datasets [20]. These approaches have shown promising results in urban sensing, mobility analysis, and environmental monitoring scenarios [21,22].

However, handling missing observations remains a major challenge in sensor networks. Traditional imputation techniques, such as statistical interpolation or simple forward-filling strategies, often fail to capture the temporal dynamics of irregularly sampled environmental data. For this reason, several studies have proposed learning-based approaches in which neural networks are used to reconstruct or infer missing information directly from the temporal structure of the data. Recurrent architectures, in particular, have proven effective for modeling temporal dependencies and identifying anomalous patterns in meteorological time series [23].

A widely adopted approach for handling missing data in time series is the Gated Recurrent Unit with Decay (GRU-D) framework, which introduces time-dependent decay mechanisms to model the influence of past observations as a function of the elapsed time since the last measurement [24]. In this formulation, the contribution of previously observed values progressively decreases as the time gap increases, while statistical baselines derived from historical data are used to guide the reconstruction of missing values. More recently, these temporal decay concepts have been extended to dynamic graph learning frameworks, where missing data may affect not only node features but also the underlying graph structure. In such settings, hybrid models combining graph reconstruction mechanisms with temporal sequence modeling have been proposed to handle incomplete graph snapshots caused by communication failures or sensor outages [25,26].

These developments highlight the growing relevance of graph-based deep learning frameworks for modeling complex sensor networks characterized by spatial dependencies, temporal irregularity, and incomplete observations. However, the joint modeling of physical spatial topology, temporal irregularity, and fault-induced missing data remains a largely open problem in distributed environmental sensor network learning. Motivated by these considerations, the present work introduces a spatio-temporal graph-based autoencoder designed for the reconstruction of missing environmental observations in vineyard sensor networks. The proposed framework explicitly combines temporal decay-aware sequence modeling with spatial message passing over a graph reflecting the physical topology of the sensing infrastructure. By jointly learning temporal dynamics within individual sensor streams and spatial dependencies among neighboring nodes, the model aims to provide robust and physically consistent reconstruction of temperature and humidity time series under controlled conditions of data loss and irregular sampling.

## 3. Materials and Methods

### 3.1. Hardware Platform for Environmental Data Collection

The datasets considered in this study were acquired using a custom-developed wireless sensing platform for precision agriculture applications. The hardware architecture and validation procedures have been extensively described in previous contributions [7,17,23,27]; therefore, only a concise overview of the system configuration and deployment is provided.

The platform is composed of five autonomous, battery-powered sensing nodes designed for long-term operation in outdoor vineyard environments. The system was engineered with particular attention to low power consumption, reduced implementation cost through commercial off-the-shelf components, and modular design, enabling flexible and scalable deployment strategies.

**Figure 1:**
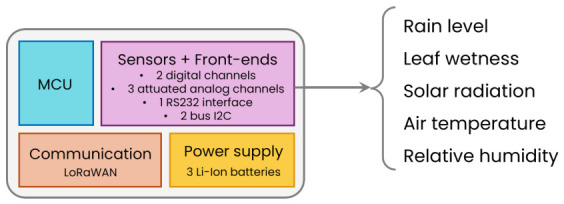
Schematic representation of the building blocks composing the realized sensor node.

Each sensing node integrates multiple environmental sensors for climatic monitoring. Air temperature and RH are measured by a digital SHT30 sensor (Sensirion, Stäfa, Switzerland), selected for its stability and compatibility with ultra-low-power embedded systems. Solar irradiance is estimated through cadmium sulfide (CdS) light-dependent resistors (Advanced Photonix, Camarillo, CA, USA), whose radiometric behavior under field conditions was characterized in previous studies [7]. Leaf wetness is detected using resistive interdigitated probes (MH-RD type) positioned to sense moisture conditions on both upper and lower leaf surfaces. Prior electrical characterization of the resistive sensors allowed impedance variations to be mapped into a discrete wetness index used for subsequent data analysis. Precipitation is monitored through an integrated tipping-bucket rain gauge, enabling site-specific rainfall measurements. The processing unit is based on an STM32L073RBT6 ultra-low-power microcontroller (STMicroelectronics, Plan-les-Ouates, Switzerland), supporting duty-cycled acquisition and communication to extend battery lifetime. Wireless connectivity is provided by a HopeRF (Shenzhen, Guangdong, China) RFM95x LoRa transceiver operating in the 868 MHz ISM band. A schematic representation of the node architecture is reported in Figure 1.

**Figure 2:**
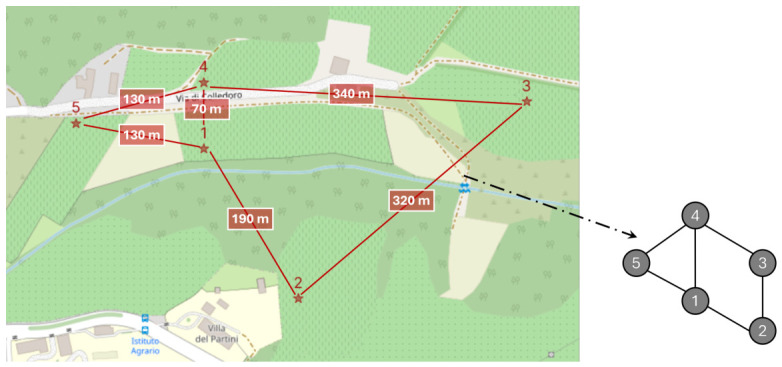
Geographical map of the sensor network deployed in the chosen test site and corresponding graph representation defined according to a spatial proximity relationship (i.e., each node is connected to its two nearest neighbors in terms of straight-line distance).

**Figure 3:**
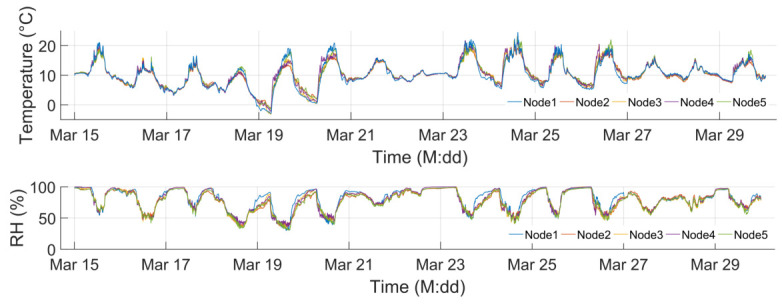
Temperature and RH temporal evolution for the five nodes (as per legend) during 15 days of tests in March 2025.

Five wireless sensor nodes were deployed at the Istituto Tecnico Agrario B. Ricasoli (Siena, Italy), as shown in Figure 2, enabling continuous monitoring of vineyard microclimatic conditions over an extended observation window spanning from July 2023 to May 2025. Measurements were acquired and transmitted every five minutes, according to a periodic sampling scheme designed to balance energy efficiency and communication reliability. The packets were then collected by an outdoor gateway installed on the main farm building and forwarded to a ChirpStack LoRaWAN network server (version 4.6.0) for storage and management. Remote access, visualization, and inspection of the acquired measurements are provided through a Grafana-based dashboard interface (version 10.2.3). While the sensing infrastructure monitored several environmental parameters, the analysis presented in this work is restricted to the air temperature and RH time series. Representative temporal profiles for these quantities are illustrated in Figure 3 for about two weeks. The temperature series exhibit the expected diurnal oscillations, with peak values occurring during daytime and minima during nighttime, while RH shows an inverse trend. The five nodes display a high degree of temporal coherence, with only minor differences attributable to site-specific environmental conditions and variability across vineyard locations. Indeed, although the sensing units were deployed within a relatively confined geographical area (inter-node distance < 500 m), they were installed in vineyard plots characterized by slightly different elevations and solar exposures, resulting in moderate climatic variability. At the same time, the spatial arrangement of the sensing nodes can induce similarities in the temporal evolution of the recorded variables, as sensors located closer to each other are likely to experience comparable environmental conditions. This observation suggests that spatial information can be exploited to support data-driven modeling of the sensor measurements. To this end, the sensing network is represented as a graph G=(V,E), where each vertex v∈V corresponds to a sensor node and edges e∈E encode relationships among sensors. In particular, the graph topology is defined according to a spatial relationship due to geographical proximity, by connecting each node to its two nearest neighbors based on the straight-line distance between sensor locations. The corresponding graph is reported in Figure 2.

### 3.2. Neural Network Architecture

**Figure 4:**
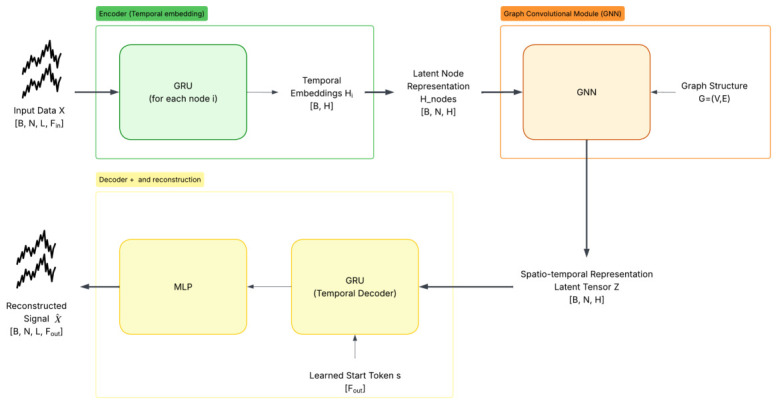
Schematic representation of the STGNN-AE architecture and data processing flow. The integration of spatio-temporal features follows a hierarchical and sequential approach. (a) Temporal Phase: the recurrent encoder independently processes each node’s time series to extract a latent embedding that condenses local temporal dynamics. (b) Spatial Phase: the resulting temporal embeddings are mapped as initial node features; the GraphSAGE module then performs message passing, aggregating neighbor information to incorporate spatial context. (c) Reconstruction: the joint latent tensor *Z*, integrating both historical memory and spatial correlation, initializes the decoder for the signal reconstruction process.

A Spatio-Temporal Graph Neural Network Autoencoder (STGNN-AE) is developed to reconstruct multivariate time series collected from interconnected sensing units, naturally represented as a graph G=(V,E). The network architecture is shown in Figure 4.

Specifically, temporal patterns are first encoded independently at the node level in order to extract compact representations of local dynamics. These node-wise embeddings are then combined through graph-based message passing, allowing spatial dependencies to be incorporated into a unified latent representation. In this way, both local temporal evolution and cross-node interactions are simultaneously captured. Moreover, message passing among nodes is performed at the level of learned temporal representations rather than raw timestep-wise observations. This design reduces the computational cost associated with performing graph operations at every time step and avoids mixing spatial information before node-specific temporal patterns have been encoded.

**Temporal Node Encoder**

The input to the model is organized as a four-dimensional tensor X$\in R^{B\times N\times L\times F_{in}}$, where *B* denotes the batch size, N=|V| the number of sensor nodes, *L* the temporal window length, and $F_{in}$ the number of measured features at each timestep. The input features are preprocessed using a time-decay imputation mechanism inspired by the GRU-D framework, and subsequently fed into the network. This allows the standard GRU to process time-aware features (including the time elapsed since the last valid observation) while maintaining high computational efficiency. More details about preprocessing are reported in Section 3.3.

For each node i∈V, the corresponding time series $x_{i}=\left\{x_{i,1},\ldots ,x_{i,L}\right\}$ is processed independently by a temporal encoder $\phi_{enc}$ based on a GRU [28]. The GRU is adopted for its ability to efficiently model sequential dependencies while mitigating vanishing gradient issues. Through the sequential update of hidden states over t∈[1,L], temporal information is progressively aggregated into a compact latent representation.

At each timestep, two gating mechanisms regulate the information flow. The update gate $z_{t}$ controls the extent to which the candidate hidden state contributes to the current hidden state, effectively balancing between retaining past information and incorporating new content. The reset gate $r_{t}$, in contrast, modulates the contribution of the previous hidden state in the computation of the candidate activation, enabling the model to selectively forget irrelevant past information.

The recurrent dynamics is defined as

$$\begin{array}{c} z_{t}=\sigma \;\left(W_{z}\cdot \left[h_{t-1},x_{i,t}\right]+b_{z}\right)\\ r_{t}=\sigma \;\left(W_{r}\cdot \left[h_{t-1},x_{i,t}\right]+b_{r}\right)\\ {\tilde{h}}_{t}=\tanh\;\left(W_{h}\cdot \left[r_{t}⊙h_{t-1},x_{i,t}\right]+b_{h}\right)\\ h_{t}=\left(1-z_{t}\right)⊙h_{t-1}+z_{t}⊙{\tilde{h}}_{t} \end{array}$$

where $b_{z}$, $b_{r}$, $b_{h}$ are bias vectors and $W_{z}$, $W_{r}$, $W_{h}$ are learnable weight matrices. After processing the entire temporal window, the final hidden state $h_{L}\in R^{H}$ is retained as the latent temporal embedding for node *i*, denoted as $H_{i}$. This embedding summarizes the temporal behavior of the sensor over the considered time horizon and serves as the input to the subsequent spatial modeling stage.

**Graph Convolutional Module**

To explicitly model the spatial relationships among sensors, a Graph Neural Network (GNN) module is incorporated into the architecture. In this work, the spatial component of the model is implemented using the GraphSAGE framework [29], an inductive graph representation learning method designed to efficiently propagate information across neighboring nodes. GraphSAGE learns node representations by aggregating information from the local neighborhood of each node and combining it with the node own features. This formulation allows the model to generalize to unseen nodes or graph structures, which is particularly advantageous in dynamic sensor networks where node availability may change over time. Formally, at layer l+1, the representation of node *v* is updated according to

$$h_{v}^{\left(l+1\right)}=\sigma \;\left(W\cdot \mathrm{concat}\left(h_{v}^{\left(l\right)},\mathrm{AGG}\;\left(\{h_{u}^{\left(l\right)}|u\in N\left(v\right)\}\right)\right)\right)$$

where N(v) denotes the set of neighbors of node *v*, W is a learnable weight matrix, and σ(·) is a nonlinear activation function. The aggregation operator AGG(·) is permutation-invariant (e.g., mean or pooling), ensuring robustness with respect to the ordering of neighboring nodes. In this work, mean aggregation is adopted due to its stability and robustness to noisy measurements.

**Spatio-Temporal Embedding in Latent Space**

Following the temporal encoding, the node-level embeddings are arranged into $H_{nodes}\in R^{B\times N\times H}$, with $H_{i}\in R^{B\times H}$ denoting the representation of node *i*. These embeddings are then processed through *m* layers of graph message passing, yielding the spatially enriched latent tensor Z:

$$Z=\mathrm{GNN}(H_{nodes},E)$$

This operation enables information exchange among spatially connected nodes, allowing each latent representation to integrate neighborhood context. As a consequence, spatial dependencies among sensors are explicitly modeled within the latent space. The resulting tensor Z jointly captures temporal dynamics, learned by the GRU encoder, and spatial correlations—modeled through graph message passing. These enriched embeddings are subsequently used to initialize and condition the decoder during the reconstruction process.

No additional graph operations are performed during decoding. This design choice reduces computational complexity while preserving the essential spatial structure encoded in the latent representation, thus ensuring suitability for practical sensor network deployments.

**Temporal Decoding and MLP Projection**

Sequence reconstruction is carried out by a decoder $\psi_{dec}$ implemented as a GRU. The decoder is initialized from the spatially enriched latent representation Z, which compactly captures both temporal and spatial information.

Let $S\in R^{B\times L\times F_{out}}$ be a tensor obtained by replicating the learned start token $s\in R^{F_{out}}$ along the temporal dimension. The decoder dynamics can then be written as:

$$H_{dec}=\mathrm{GRU}\;\left(S,Z\right)$$

Through this process, the decoder progressively reconstructs the temporal signal over the entire prediction horizon. The resulting sequence of hidden states $H_{dec}$ is finally projected back into the original feature space by means of a Multi-Layer Perceptron (MLP), producing the reconstructed signal:

$$\hat{X}=\mathrm{MLP}\left(H_{dec}\right)$$

This projection ensures that the output $\hat{X}$ matches the dimensionality of the input data, enabling direct comparison between the reconstructed and observed sensor measurements.

**Loss Function**

To improve reconstruction performance, a composite loss function is employed that combines a conventional point-wise error term with a correlation-based component. Specifically, the proposed objective integrates the Mean Absolute Error (MAE), which penalizes absolute deviations between predicted and target values, with a Pearson correlation loss designed to promote similarity in temporal trends. Correlation-based objectives have previously demonstrated effectiveness in tasks such as neural rendering and depth reconstruction [30,31], where augmented Pearson loss formulations have been shown to enhance local structural consistency between predicted and ground-truth representations. By jointly minimizing amplitude discrepancies and maximizing linear correlation, the proposed loss encourages accurate signal reconstruction while preserving the underlying temporal dynamics.

Given the predictions $\hat{X}$ and targets X, the Pearson correlation coefficient is computed along the temporal dimension after mean-centering both signals. The correlation term encourages alignment in both shape and temporal dynamics, regardless of differences in scale. The corresponding loss component is defined as

$$L_{\mathrm{Pearson}}=1-\mathrm{mean}\left(\rho \right)$$

where ρ denotes the Pearson correlation coefficient computed across the time axis. The total loss is then obtained as the weighted combination

$$L_{\mathrm{total}}=\alpha L_{\mathrm{MAE}}+\left(1-\alpha \right)L_{\mathrm{Pearson}}$$

where α∈[0,1] controls the trade-off between amplitude accuracy and temporal trend alignment. This formulation allows the model to simultaneously minimize reconstruction error while preserving the temporal structure of the signals, which is particularly important in sensor-based time series where relative dynamics may carry significant information.

**Denoising Training Strategy**

The proposed model is trained to reconstruct clean signals from deliberately corrupted inputs. This training strategy encourages the latent representation to capture the intrinsic structure of the data rather than memorizing individual observations, thereby improving robustness to noise and missing measurements.

Input corruption is performed through a structured masking procedure applied to the input tensor X. For each element in the batch, a single node *u* is randomly selected from the set of available nodes. A temporal mask vector is then sampled according to a Bernoulli distribution with probability *p*, which determines the timesteps to be corrupted.

For each selected timestep, the masking operation is applied simultaneously across all feature channels of the chosen node. The corresponding entries are replaced with a constant sentinel value *s* (set to −4.0 in our implementation), representing missing or corrupted measurements, while all other nodes and unmasked timesteps remain unchanged. This procedure forces the model to infer the corrupted values by exploiting both temporal context and spatial information from neighboring nodes.

The complete data corruption procedure is summarized in Algorithm 1.
**Algorithm 1** Structured Node-wise Temporal Masking**Require:** Input batch $X\in R^{B\times N\times L\times F_{in}}$, masking probability *p*, sentinel value *s*

**Ensure:**
Corrupted batch $\tilde{X}$
  1:$\tilde{X}\leftarrow X$  2:**for** b=1,…,B **do**  3:   Sample node index n∼U(1,N)  4:   Sample mask vector $m\in {\left\{0,1\right\}}^{T}$ with $P(m_{t}=1)=p$  5:   **for** *t* such that $m_{t}=1$ **do**  6:     $\tilde{X}\left[b,n,t,:\right]\leftarrow s$  7:   **end for**  8:**end for**  9:**return** $\tilde{X}$


This node-wise and time-selective corruption strategy simulates missing-data scenarios in sensor networks, where individual sensor nodes may experience temporary data gaps due, for example, to radio transmission issues. By training the model to recover the original signal from these partially masked inputs, the architecture learns to exploit both temporal continuity and spatial correlations across nodes, thereby improving generalization and reconstruction performance.

### 3.3. Dataset Preprocessing

The raw measurements generated by each node were transmitted via LoRaWAN and stored at the server side as discrete uplink events. Each event contains the reception timestamp, the unique node identifier, and the associated sensor readings. This packet-based structure guarantees internal temporal consistency among variables acquired within the same transmission cycle. However, the five nodes operate autonomously and are not synchronized. Although configured for a nominal sampling interval of five minutes, the effective inter-arrival times exhibit variability. Consequently, the raw datasets consist of irregularly spaced time series that cannot be directly compared across nodes. In addition, each node may experience distinct failure mechanisms, including sensor-specific malfunctions, power instabilities, or communication-related packet losses intrinsic to LPWAN operation. The raw dataset collected from the vineyard nodes exhibits an average missing rate of approximately 10% across all nodes, primarily attributable to communication and hardware failures.

Therefore, prior to model development, the pre-processing phase was designed to ensure temporal comparability and data reliability. First, the five independent sequences were mapped onto a common temporal reference grid to obtain aligned time series suitable for cross-node analysis. Subsequently, data quality control procedures were applied. Time intervals corresponding to verified sensor or node anomalies were removed from all five time series, even when numerical readings were present but considered unreliable. To avoid temporal leakage while preserving seasonal variability, the dataset was split using a month-wise stratified strategy. Each monthly segment was divided chronologically into training (50%), validation (20%), and test (30%) subsets. This approach ensures that sliding windows do not cross discontinuous temporal segments while maintaining representative seasonal patterns in all subsets. For the 3 h temporal configuration (L=36), the process generated 88,582 training samples, 1149 validation samples, and 1495 test samples. When the window length was extended to 12 h (L=144), the dataset comprised 69,256 training samples, 277 validation samples, and 482 test samples. The high density of training samples is a direct result of the nearly total overlap (stride = 1) employed to enhance the model’s ability to learn complex spatio-temporal dependencies. Conversely, the lower sample counts in the validation and test sets reflect the use of larger strides (1/6 of the window length for validation) and non-overlapping segments for testing, respectively, ensuring that the final performance evaluation is conducted on strictly independent temporal windows. Temporal discontinuities were analyzed to distinguish short-term transmission losses from prolonged outages. Short-duration gaps were reconstructed using a GRU-D-inspired imputation strategy [24].

**GRU-D-inspired Imputation and Time-Dependent Baseline**

Missing values were handled using a GRU-D-based imputation mechanism that combines temporal decay with a time-dependent baseline. Specifically, the baseline term $\mu_{f}\left(t\right)$ was defined as the mean value of feature *f* conditioned on both the month and the hour associated with each timestamp; i.e., $\mu_{f}\left(\mathrm{month},\mathrm{hour}\right)$. Each timestamp was converted into its corresponding month (1–12) and hour (0–23), and feature-wise averages were computed across all observed samples within each month-hour combination, yielding 288 temporal bins (12 months × 24 h). For a given timestamp *t*, when sufficient observations were available in the corresponding bin, the baseline $\mu_{f}\left(t\right)$ was assigned as the mean value of that bin.

To ensure robustness under sparse data conditions, a hierarchical fallback strategy was adopted. If the month–hour bin contained no observations, the algorithm reverted to the hourly mean aggregated across all months. If this was also unavailable, the monthly mean was used. As a final fallback, the global feature mean over the entire dataset was applied.

Missing values were then reconstructed using an exponential decay formulation:

$$x_{\mathrm{imp}}\left(t,f\right)=\gamma \left(t,f\right)\cdot x_{\mathrm{last}}\left(t,f\right)+\left(1-\gamma (t,f)\right)\cdot \mu_{f}\left(t\right)$$

where $x_{\mathrm{last}}\left(t,f\right)$ denotes the most recent observed value and γ(t,f)=exp(−λΔt(t)) modulates the influence of past observations according to the elapsed time Δt(t) since the last valid measurement. The decay rate λ was determined using a fixed half-life criterion of 4 h, ensuring that the contribution of past observations decreases progressively while avoiding excessively long memory effects. The selection of λ = 4 h was informed by the autocorrelation analysis of the field data. This window effectively models the stability period of vineyard climatic variables before significant shifts occur during diurnal transitions. This choice aligns with established stochastic models for air temperature, which characterize the variable as a first order auto-regressive process with hourly persistence coefficients typically ranging between 0.80 and 0.90 [32]. At this scale, the 4 h threshold balances the persistence of past observations with the need to avoid the influence of outdated historical data. This strategy enables the model to exploit seasonal and diurnal regularities while progressively attenuating outdated information. The adoption of a GRU-D-inspired strategy was motivated by the limitations in the presence of missing observations of standard recurrent architectures, that do not take into account the time elapsed since the last valid observation. As a result, missing data must typically be filled before training, without providing the model with information about the reliability or temporal age of the imputed values. GRU-D-inspired was specifically introduced to address this issue by incorporating a temporal decay factor that progressively reduces the contribution of outdated measurements. This mechanism is particularly suitable for time series derived from environmental sensor networks, where the duration of a data gap carries meaningful information about the reliability of past observations.

In addition, cyclic time-of-day encodings (sine and cosine components) were derived from the timestamps to capture daily periodicity. For each timestamp *t*, the final feature vector was constructed as:

$$\left[x_{\mathrm{imp}}\left(t,f\right),\;\Delta t\left(t\right),\;\sin\left(\frac{2\pi \cdot hour(t)}{24}\right),\;\cos\left(\frac{2\pi \cdot hour(t)}{24}\right)\right]$$

Following feature construction, the processed time series were transformed into samples using a sliding-window approach. Each input window had length *L* (lookback), and the prediction horizon was set to one step ahead. Different strides were adopted across splits to control window overlap: a high overlap in the training set to increase sample density, reduced overlap in validation, and no overlap in the test set to prevent information leakage. Finally, windows with insufficient true data availability were removed by enforcing a minimum mask-coverage threshold of 50%. This decision serves as a strategic compromise: it ensures that each temporal window contains enough “ground truth” to provide a meaningful training signal, while still allowing the model to learn from realistic, partially incomplete sequences. By discarding windows where more than half of the target values were missing, we protect the training process from the high uncertainty associated with excessively sparse data, thereby ensuring a minimum quality of the learned spatio-temporal representations.

The data are subsequently normalized using z-score normalization, where each feature is standardized by subtracting the mean and dividing by the standard deviation computed on the training set.

## 4. Experimental Results

The training procedure was designed to reconstruct a single sensor variable at a time. In particular, the model was trained alternatively to reconstruct either temperature or humidity measurements, allowing the reconstruction performance to be evaluated independently for the two sensing modalities.

The entire framework was implemented in *PyTorch 2.9.0*, enabling efficient training and experimentation with GPU acceleration. All experiments were conducted on a workstation equipped with an NVIDIA GeForce RTX 4090.

An initial hyperparameter optimization phase was performed with L=36 (3 h) using the Optuna framework [33], in order to identify the optimal hyperparameter configuration. The selected hyperparameters are summarized in Table 1. Optuna was employed to efficiently explore the hyperparameter space and identify the combination of architectural and training parameters that minimizes the validation loss, computed over the complete duration of the input sequence. No additional metrics were included as independent optimization objectives in the hyperparameter search, since the optimized loss already jointly accounts for amplitude reconstruction errors and temporal shape consistency.

The hyperparameter search included the dimension of the latent representation *h*, the number of layers in the GRU encoder module, the number of message-passing layers in the GNN component, and the weighting parameter α used in the loss function. The decoder uses a single GRU layer, while the final projection module is implemented as an MLP composed of three layers with ReLU activations.

Once the optimal configuration was identified, the hyperparameters were fixed and no further tuning was performed. The selected configuration was then trained using a *k*-runs training strategy with k=10 using different random seeds, in order to mitigate the influence of random initialization and training stochasticity. The input window length *L* was set to two values, L=36 and L=144, corresponding to temporal spans of 3 h and 12 h, respectively. Subsequent experiments were conducted using both values of *L* in order to assess the model under different temporal contexts. In each run, the model was trained using the corruption procedure described in Algorithm 1, which applies structured node-wise temporal masking to the input signals as part of the denoising training paradigm. The corruption probability during training was fixed at p=0.5. This value was deliberately chosen to be significantly higher than the average raw missing rate (approx. 10%) to ensure the model could handle extreme data loss scenarios, thereby improving the overall robustness and generalization capability of the network.

The results of the *k*-run experiments are reported in Table 2 as the mean and standard deviation across ten independent runs, using the reconstruction loss computed over the entire input sequence.

After completing the ten-run evaluation and to further assess the robustness of the learned representation, the model was evaluated on the test set under varying levels of input corruption. Specifically, the structured node-wise temporal masking procedure described in Algorithm 1 was applied using corruption probabilities p∈{0.1,0.3,0.7,0.9}. This analysis enables the evaluation of the model’s ability to reconstruct signals under increasing levels of artificially masked missing data.

**Figure 5:**
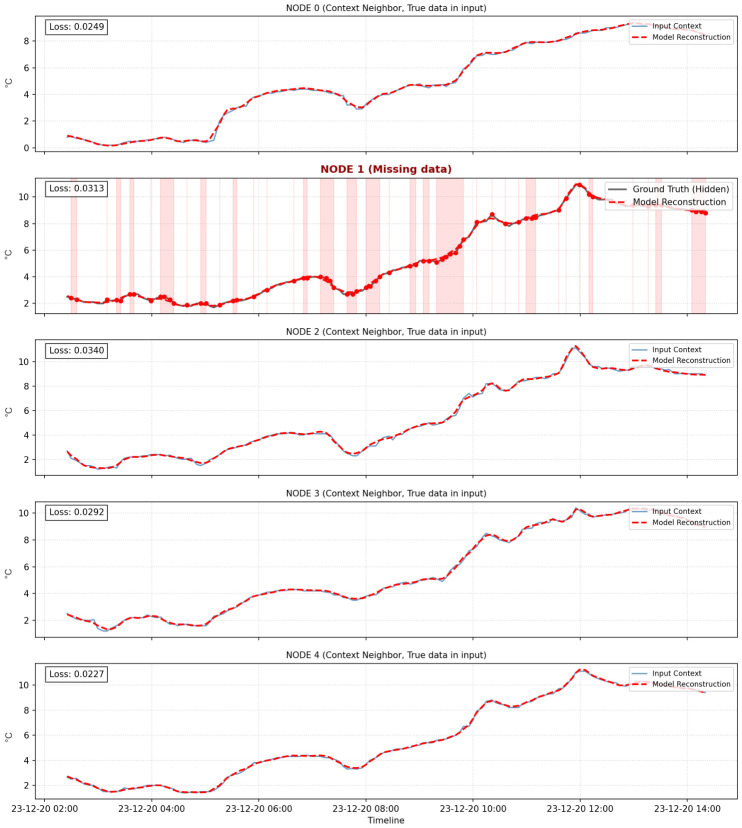
Example of the temperature signal reconstruction for L=36 as per legend. The shaded regions indicate artificially masked intervals introduced for validation, where the model must infer the missing values; red dots denote the corresponding ground-truth input points that were masked and used only for comparison. Neighboring nodes provide contextual information to support the reconstruction.

**Figure 6:**
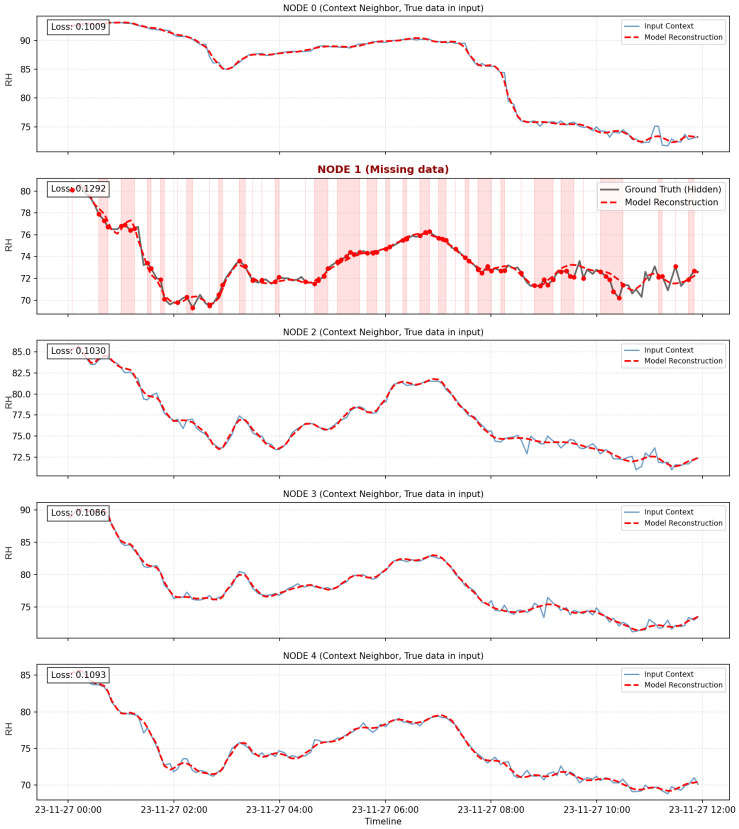
Example of the humidity signal reconstruction for L=36 as per legend. The shaded regions indicate artificially masked intervals introduced for validation, where the model must infer the missing values; red dots denote the corresponding ground-truth input points that were masked and used only for comparison. Neighboring nodes provide contextual information to support the reconstruction.

**Figure 7:**
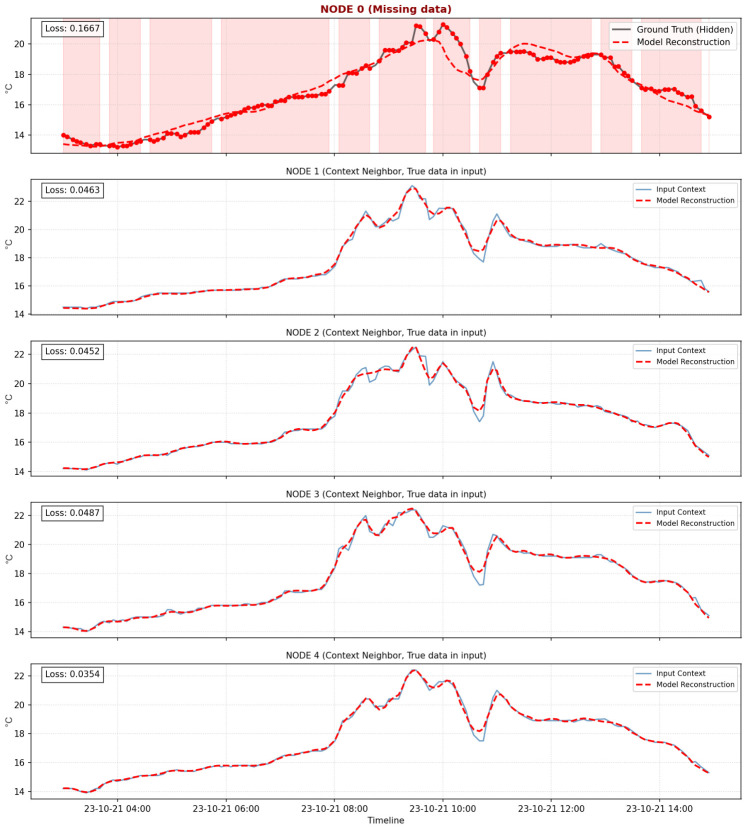
Example of the temperature signal reconstruction for L=144 as per legend with p=0.9. The shaded regions indicate artificially masked intervals introduced for validation, where the model must infer the missing values; red dots denote the corresponding ground-truth input points that were masked and used only for comparison. Neighboring nodes provide contextual information to support the reconstruction.

Figure 5 and Figure 6 present examples of signal reconstruction for temperature and humidity sensors, respectively, using a window length L=36. In Figure 7, the results for a more challenging configuration (i.e., p=0.9 and L=144) are reported for the temperature time series. Specifically, one node is partially corrupted with corruption probability p=0.5 or p=0.9, while the original observed values are retained as ground truth for comparison and the neighboring non-corrupted nodes provide contextual information to the model. The plots illustrate how the proposed spatio-temporal architecture is able to accurately reconstruct the masked measurements by jointly exploiting the temporal dynamics of the corrupted node and the spatial information propagated through the sensor network.

**Figure 8:**
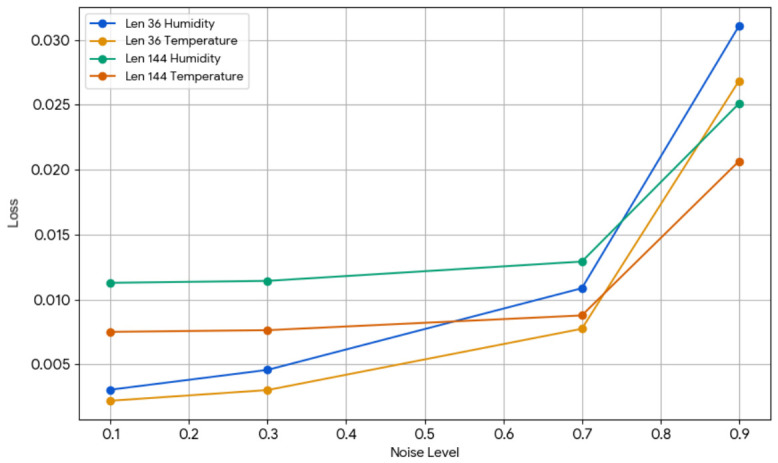
Reconstruction loss across varying corruption probabilities *p*.

The detailed results for p∈{0.1,0.3,0.7,0.9} are reported in Table 3 and Figure 8. Performance was measured using the same loss function adopted during training, computed over the entire input sequence, ensuring consistency between the training objective and the evaluation metric.

## 5. Discussion and Perspectives

### 5.1. Results Discussion

The experimental results provide several insights into the behavior of the proposed spatio-temporal reconstruction framework under missing-data conditions.

Table 3 and Figure 8 report the reconstruction performance of the model under different corruption levels, illustrating the relationship between the corruption probability and the reconstruction loss. Overall, reconstruction performance tends to degrade as the corruption probability *p* increases, although the composite reconstruction loss does not follow a strictly monotonic trend across all tested configurations. Nevertheless, as shown in Table 3, the model maintains stable performance under moderate corruption levels (*p* ≤ 0.7), demonstrating its ability to recover missing information by leveraging both temporal continuity and spatial dependencies across neighboring sensing nodes.

This robustness is further validated by the non-normalized MAE values, which provide a clear measure of the model’s precision in physical units. Both the reconstruction loss and the MAE are computed over the entire input sequence, rather than being restricted to the artificially masked values. For the temperature, the reconstruction error remains remarkably low: considering L=36, it ranges from 0.018 °C (p=0.1) to a maximum of 0.133 °C at p=0.9. When the window size is increased to L=144, the model starts from a higher baseline (0.099 °C) but maintains a stable error profile, peaking at 0.226 °C under extreme masking conditions. A similar behavior is observed for humidity, where the MAE scales from 0.074% to 0.425% for L=36, and reaches 0.783% for L=144 at the highest corruption level.

A key observation emerging from the experimental results concerns the role of spatial information in stabilizing the reconstruction process. Even when a significant portion of the temporal series of a node is masked, the model is still able to infer plausible signal dynamics by exploiting information propagated through the graph structure. This suggests that the latent representations learned by the GraphSAGE module effectively encode correlations between nodes induced by shared environmental conditions. In practical terms, this confirms that geographically proximate sensors can provide meaningful contextual information for reconstructing missing microclimatic measurements. The comparison between the two temporal window configurations (L=36 and L=144) reveals an additional trade-off between temporal context and reconstruction stability. Shorter windows, corresponding to approximately three hours of observations, consistently yield lower reconstruction losses than longer twelve-hour windows for p≤0.7. This result may be explained by the quasi-smooth and locally predictable evolution of temperature and humidity signals over short horizons. Conversely, longer temporal contexts introduce increased variability due to changes in site-specific climatic effects. As a consequence, the reconstruction task becomes more challenging, especially when large portions of the signal are corrupted.

Interestingly, the degradation pattern observed at high masking probabilities (p=0.9) highlights the limits of spatial compensation mechanisms. When nearly all temporal information from the corrupted node is removed, the model increasingly relies on neighboring nodes, whose dynamics, although correlated, are not identical. This leads to larger reconstruction errors and indicates that spatial dependencies alone are insufficient to fully recover node-specific signal characteristics under extreme data loss conditions. Such behavior is consistent with the physical heterogeneity of vineyard environments, where elevation, solar exposure, and local canopy structure can induce persistent microclimatic differences.

In the extreme corruption scenario (p=0.9), the configuration with L=144 achieves a lower composite reconstruction loss than L=36, while exhibiting a higher unnormalized MAE. This suggests that the longer temporal context improves the preservation of global temporal patterns, as captured by the correlation component of the loss, while not improving point-wise accuracy. Under very high masking levels, the amount of reliable information from the corrupted node becomes extremely limited, reducing the effectiveness of short-term temporal dependencies. In this condition, the reconstruction process benefits from a wider temporal receptive field, which provides additional historical data and allows the model to better exploit slow-varying environmental trends such as diurnal cycles and gradual atmospheric transitions. Furthermore, longer windows enable the latent representation to capture more global signal regularities, partially compensating for the reduced availability of local temporal information. As a consequence, the model can leverage broader temporal patterns to stabilize the reconstruction, leading to a slightly improved performance compared to shorter windows under extreme data loss.

The qualitative examples reported in Figure 5, Figure 6 and Figure 7 further support these quantitative findings. The reconstructed series preserve the smooth temporal evolution and the relative amplitude variations of the ground-truth signals, even across consecutive masked intervals. This suggests that the composite loss function, combining MAE minimization with a correlation-based objective, effectively encourages the model to learn both point-wise accuracy and global trend consistency. In environmental sensing applications, this property is particularly desirable, as preserving diurnal dynamics and relative fluctuations is often more informative than minimizing instantaneous errors alone. Another important aspect concerns the denoising training paradigm adopted in this work. By systematically exposing the model to structured node-wise temporal masking during training, the network learns to infer missing observations from partially corrupted inputs, resulting in improved robustness at inference time.

A further insight can be obtained by comparing the reconstruction performance achieved for temperature and RH signals. As shown in Figure 8 and Table 3, humidity consistently exhibits higher reconstruction losses than temperature across all masking probabilities and temporal window configurations, suggesting that it collects less reliable information from neighboring nodes. To support this interpretation, the characteristics of the two variables were further analyzed. First, pairwise Pearson correlation coefficients were computed among the five sensing nodes. The analysis confirmed that both variables exhibit spatial coherence across the monitored vineyard, but temperature shows stronger and more uniform inter-node correlation. This indicates that temperature provides a higher degree of spatial redundancy for the graph-based reconstruction process, while humidity exhibits weaker and more heterogeneous spatial synchrony. Second, the temporal variability of the two signals was quantified using the mean absolute difference between consecutive days computed on the daily mean series. The normalized day-to-day fluctuation of RH was found approximately 2.5 times higher than that of temperature, confirming that RH is less smooth and more affected by short-term variability.

These quantitative indicators are consistent with the physical mechanisms governing the two variables. Air temperature in outdoor environments is mainly driven by solar radiation and atmospheric heat exchange processes, which tend to generate smooth daily and seasonal cycles and coherent spatial patterns over short distances. RH, instead, depends on a broader set of local and transient processes, including evapotranspiration, canopy wetness, condensation, rainfall occurrence, and localized airflow patterns. These mechanisms can introduce sharper fluctuations and stronger node-specific variations, especially in vineyard plots characterized by different exposure, canopy structure, or terrain conditions. As a consequence, the smoother temporal evolution of temperature facilitates its temporal encoding, while its stronger inter-node correlation makes spatial information from neighboring sensors highly informative. Conversely, the higher temporal fluctuation and weaker spatial synchrony of RH reduce the predictive value of both the local temporal history and the spatial context propagated through the graph.

Another aspect concerns the metrological characteristics of the adopted sensing channel. According to the nominal specifications of the SHT30-based sensor, the accuracy is approximately ±0.2 °C for temperature and ±2% for RH. The RH channel is therefore intrinsically associated with a larger measurement uncertainty. Nevertheless, the unnormalized MAE values obtained for RH remain below the nominal RH accuracy in the tested configurations. Consequently, the reconstruction error remains compatible with the practical uncertainty range of the sensing platform.

### 5.2. Ablation Studies

To rigorously justify the architectural necessity of each core component within the proposed framework, an ablation study was conducted. First we evaluated the performance degradation when removing the spatial graph neural network module, thereby forcing the architecture to rely solely on the temporal representations for reconstruction. The temporal-only baseline was compared against the full STGNN-AE using a window length of L=144 under two distinct data-loss scenarios: standard random corruption level (p=0.5) and complete node missing data (p=1.0), where the entire temporal sequence of a randomly selected node was fully masked. The quantitative results are summarized in Table 4.

The empirical evidence indicates that while the temporal-only model achieves comparable performance under moderate, non-contiguous data gaps (p=0.5), it undergoes severe performance degradation under extreme data-loss conditions (p=1.0). When an entire node sequence is missing, the lack of local historical context causes the temporal-only baseline to collapse, yielding high reconstruction losses (0.174 for temperature and 0.156 for humidity). In contrast, the integration of the GraphSAGE module stabilizes the system, reducing the reconstruction loss to 0.022 and 0.025, respectively. This demonstrates that the spatial message-passing mechanism effectively extracts contextual structural redundancies from neighboring sensors to reconstruct localized signals during total data missing.

The second comparative analysis evaluates the proposed STGNN-AE against two standard univariate imputation baselines commonly used in time-series reconstruction: linear interpolation and forward filling. These deterministic methods were tested on the same test set under identical random masking scenarios. For ease of comparison, Table 5 also reports the STGNN-AE MAE values previously presented in Table 3. As shown in Table 5, their relative performance changes markedly as the severity of data loss increases.

At low corruption levels (e.g., p=0.1 and p=0.3), linear interpolation yields lower or comparable MAE. This behavior is expected: when missing data intervals are extremely short, the smooth thermodynamic evolution of microclimatic variables can be accurately approximated by simply connecting adjacent observations. However, as the corruption ratio *p* increases—simulating prolonged sensor failures—the univariate baselines experience a drastic degradation in performance due to their inability to model non-linear diurnal cycles over long gaps.

Under extreme masking conditions (p=0.9), the STGNN-AE significantly outperforms both baselines. For instance, with a temporal window of L=36, the STGNN-AE achieves a temperature MAE of 0.133, whereas linear interpolation and forward filling errors increase to 0.627 and 0.785, respectively. Comparable results are observed for relative humidity and for L=144. This outcome definitively confirms the value of the spatial module: while naïve temporal interpolation is adequate for negligible and isolated packet loss, the STGNN-AE architecture becomes essential in severe blackout scenarios, as it successfully extracts compensatory physical correlations from neighboring nodes when a specific sensor’s temporal history is completely compromised.

### 5.3. Runtime Performance

To assess the practical feasibility of the proposed framework in real-world IoT scenarios, we evaluated its computational overhead and inference latency. The STGNN-AE is a lightweight architecture comprising approximately 277.4k parameters, with a total memory footprint of 1.06 MB (FP32). The parameter distribution is dominated by the two-layer GRU encoder (54%), while the spatial GNN module accounts for only 24% of the total complexity. The benchmark results show that the single-shot inference latency on a standard CPU is low, ranging from 2.3 ms (L=36) to 8.2 ms (L=144). Considering the 5-min transmission interval of the sensor nodes, the computational cost of real-time reconstruction is negligible, allowing for seamless integration into operational decision support systems without requiring high-performance hardware.

### 5.4. Limitations and Future Directions

Despite the promising reconstruction performance, some limitations should be considered when interpreting the results. The relatively small size of the sensor network may limit the variety of spatial dependency patterns observed during training. As a consequence, the graph-based representations learned by the model may capture only a restricted set of inter-node interactions, reflecting the relatively strong spatial coherence of the monitored climatic conditions. Moreover, the graph topology is static and defined solely based on geographical proximity, which may not fully capture the complexity of environmental interactions among nodes. Real environmental interactions are often governed by time-varying processes that are not strictly distance-dependent and that cannot be adequately represented by a fixed graph topology (e.g., transient cloud coverage, canopy growth dynamics, terrain-induced airflow, topographical barriers). Consequently, the spatial message-passing mechanism may not always reflect the true instantaneous coupling between sensing locations.

The choice of a static K=2 nearest-neighbor topology was justified through extensive comparative evaluations. Preliminary tests using a correlation-based strategy—connecting nodes with the highest Pearson coefficients—resulted in redundant feature extraction due to the high temporal coherence of the vineyard microclimate (>0.9). Furthermore, increasing connectivity to K=3 or K=4 led to an “overly connected” graph for such a small-scale network (five nodes), causing a dilution of node-specific information during message passing. We also evaluated a fully connected configuration using a GATv2 architecture to infer attention weights, which did not outperform the sparser K=2 GraphSAGE model. This suggests that for climatic monitoring at this scale, a localized distance-based graph provides the optimal balance between spatial context and signal integrity, avoiding the over-smoothing typical of denser architectures.

Furthermore, although the temporal masking provides an effective and realistic approximation of communication losses or temporary sensor faults, it may not fully reproduce the diversity of failure mechanisms encountered in real deployments. For instance, long-term sensor drifts or correlated outages affecting multiple nodes simultaneously may generate reconstruction scenarios that differ from the corruption patterns simulated during training. Exploring more heterogeneous and physically informed corruption models could therefore improve the robustness of the learned representations.

Another relevant aspect, not yet explored, concerns the behavior of the proposed model under extreme or adverse climatic conditions. The dataset analyzed in this study reflects real vineyard climatic variability over the considered monitoring period; however, it did not include clearly identified extreme events. This limitation is relevant because extreme climatic events may alter the assumptions exploited by the reconstruction framework since the environmental signals may change more abruptly and become more spatially heterogeneous. In such cases, reconstruction uncertainty may increase, especially when the missing values occur during the most dynamic phase of the event. At the same time, adverse field conditions may indirectly affect the sensing infrastructure by increasing packet losses, communication interruptions, or temporary node malfunctions. From this perspective, the high-corruption experiments considered in this work, particularly the case p=0.9, can be interpreted as a stress-test of the model under severe data unavailability. However, these experiments simulate missing-data conditions rather than specific meteorological phenomena, and cannot replace a dedicated validation on documented climatic events.

Finally, the experimental evaluation focuses on reconstructing individual variables independently. Although this design simplifies the analysis and allows clearer interpretation of the reconstruction behavior for temperature and RH, environmental processes are inherently multivariate. Joint reconstruction of multiple correlated variables could enable the model to exploit additional cross-feature dependencies, potentially improving robustness under severe data loss conditions. Future work could therefore investigate fully multivariate spatio-temporal reconstruction frameworks capable of capturing both inter-node and inter-variable interactions.

## 6. Conclusions

In this work, a spatio-temporal graph-based autoencoder has been proposed for the reconstruction of missing environmental data in vineyard sensor networks. The model integrates temporal sequence modeling through a GRU encoder coupled with a decay-based imputation mechanism inspired by the GRU-D framework with spatial message passing based on the GraphSAGE network, enabling the joint exploitation of temporal dynamics and inter-node correlations.

The experimental evaluation conducted on real-world vineyard data demonstrates that the proposed approach provides robust reconstruction performance under controlled missing-data conditions designed to approximate temporary sensor or communication losses. The results confirm that, even when 90% of the data is corrupted, the network effectively exploits correlations between nodes to reconstruct missing values with high accuracy, keeping average errors consistently below 0.3 °C for temperature and 1% for humidity. This behavior is particularly relevant in vineyard monitoring scenarios, where short-term data losses are common due to communication instability or temporary sensor malfunction. The ability to provide reliable reconstructions from sparse observations ensures the continuity of data streams, which is necessary for precise agro-climatic modeling and decision support systems.

The analysis further highlights a trade-off between temporal context and reconstruction accuracy. Shorter temporal windows yield improved performance in standard conditions due to the smoother and more locally predictable behavior of environmental signals. Conversely, longer temporal contexts become advantageous under extreme data loss scenarios, where broader temporal patterns and slow-varying dynamics provide additional information to compensate for the lack of local observations. Additionally, the comparison between temperature and RH reconstruction reveals that the physical characteristics of the monitored variables significantly impact model performance. Temperature, characterized by smoother dynamics and higher spatial coherence, is reconstructed more accurately than humidity, which exhibits higher variability and weaker inter-node correlation.

To conclude, the proposed framework represents a promising solution for improving data reliability in distributed agricultural monitoring systems, with potential implications for applications such as disease prediction, irrigation management, and anomaly detection. Future research directions include the development of adaptive graph construction strategies capable of capturing time-varying spatial dependencies, the extension of the framework toward multivariate reconstruction models that jointly exploit cross-variable interactions, and the validation of the approach on larger and more heterogeneous sensor networks. In addition, future studies should include event-wise evaluations under both ordinary climatic conditions and agronomically critical events, in order to better assess the robustness of the model in real operational scenarios. These extensions may further improve the generalization capability and practical applicability of spatio-temporal graph-based models in real-world environmental monitoring scenarios.

## Figures and Tables

**Table 1:** Hyperparameters selected by Optuna framework.

Parameter	Value
latent space dimension	128
layers of GRU	2
layers of GNN	2
dropout	0.0
learning rate	2 × $10^{-4}$
batch size	32
loss weight (α)	0.6

**Table 2:** Ten-run experiment results.

Sensor	Window Length (*L*)	Reconstruction Loss (Mean ± Std)
Temperature	36 (3 h)	0.0020 ± 0.0002
Temperature	144 (12 h)	0.0082 ± 0.0003
Humidity	36 (3 h)	0.0024 ± 0.0002
Humidity	144 (12 h)	0.0121 ± 0.0004

**Table 3:** Reconstruction performance as a function of different corruption probabilities *p*. The table shows the training loss and the non-normalized MAE, expressed in degrees Celsius (°C) for temperature and in percentage (%) for humidity.

Temperature				Humidity			
Window L	Noise p	Loss	MAE [°C]	Window L	Noise p	Loss	MAE [%]
36	0.1	0.002	0.018	36	0.1	0.003	0.074
	0.3	0.003	0.022		0.3	0.005	0.095
	0.5	0.002	0.028		0.5	0.002	0.122
	0.7	0.008	0.042		0.7	0.011	0.169
	0.9	0.027	0.133		0.9	0.031	0.425
144	0.1	0.008	0.099	144	0.1	0.011	0.432
	0.3	0.008	0.101		0.3	0.011	0.437
	0.5	0.002	0.104		0.5	0.012	0.448
	0.7	0.009	0.115		0.7	0.013	0.484
	0.9	0.021	0.226		0.9	0.025	0.783

**Table 4:** Ablation study results isolating the impact of the GNN spatial module on reconstruction performance (L=144).

Variable	Corruption Level (*p*)	Loss (Without GNN)	Loss (With GNN)
Temperature	0.5	0.008	0.008
	1.0	0.174	0.022
Humidity	0.5	0.019	0.012
	1.0	0.156	0.025

**Table 5:** MAEcomparison of STGNN-AE with linear interpolation and forward filling for temperature and relative humidity.

Temperature					Humidity				
*L*	*p*	MAE [°C]			*L*	*p*	MAE [%RH]		
		STGNN-AE	Linear	Forward			STGNN-AE	Linear	Forward
	0.1	0.018	0.009	0.017		0.1	0.074	0.052	0.076
	0.3	0.022	0.034	0.064		0.3	0.095	0.171	0.260
36	0.5	0.028	0.072	0.136	36	0.5	0.122	0.331	0.522
	0.7	0.042	0.152	0.270		0.7	0.169	0.612	0.971
	0.9	0.133	0.627	0.785		0.9	0.425	2.093	2.598
	0.1	0.099	0.009	0.017		0.1	0.432	0.051	0.076
	0.3	0.101	0.032	0.063		0.3	0.437	0.166	0.260
144	0.5	0.104	0.066	0.137	144	0.5	0.448	0.313	0.526
	0.7	0.115	0.131	0.280		0.7	0.484	0.552	1.007
	0.9	0.226	0.398	0.822		0.9	0.783	1.438	2.733

## Data Availability

The raw data supporting the conclusions of this article will be made available by the authors on request.

## Abbreviations

The following abbreviations are used in this manuscript:

AE	Autoencoder
CdS	Cadmium Sulfide
DAE	Denoising Autoencoder
DCRNN	Diffusion Convolutional Recurrent Neural Network
GNN	Graph Neural Network
GraphSAGE	Graph Sample and Aggregate
GRU	Gated Recurrent Unit
GRU-D	Gated Recurrent Unit with Decay
IoT	Internet of Things
ISM	Industrial, Scientific and Medical
LoRaWAN	Long Range Wide-Area Network
LPWAN	Low-Power Wide-Area Network
LSTM	Long Short-Term Memory
MAE	Mean Absolute Error
MCU	Microcontroller Unit
ML	Machine Learning
MLP	Multi-Layer Perceptron
ReLU	Rectified Linear Unit
RH	Relative Humidity
STGCN	Spatio-Temporal Graph Convolutional Network
STGMAE	Spatio-Temporal Graph Masked Autoencoder
STGNN	Spatio-Temporal Graph Neural Network
WSN	Wireless Sensor Network

## References

[1] Wolfert S., Ge L., Verdouw C., Bogaardt M.J. (2017). Big Data in Smart Farming—A review. Agric. Syst..

[2] Rouxinol M.I., Martins M.R., Barroso J.M., Rato A.E. (2023). Wine grapes ripening: A review on climate effect and analytical approach to increase wine quality. Appl. Biosci..

[3] Mowla M.N., Mowla N., Shah A.F.M.S., Rabie K.M., Shongwe T. (2023). Internet of Things and Wireless Sensor Networks for Smart Agriculture Applications: A Survey. IEEE Access.

[4] Mansouri T., Sadeghi Moghadam M.R., Monshizadeh F., Zareravasan A. (2023). IoT data quality issues and potential solutions: A literature review. Comput. J..

[5] Decorte T., Mortier S., Lembrechts J.J., Meysman F.J., Latré S., Mannens E., Verdonck T. (2024). Missing value imputation of wireless sensor data for environmental monitoring. Sensors.

[6] Choi C., Jung H., Cho J. (2021). An ensemble method for missing data of environmental sensor considering univariate and multivariate characteristics. Sensors.

[7] Cappelli I., Parri L., Tani M., Vignoli V., Fort A. Pervasive Monitoring in the Context of Precision Agriculture: Using Low-Cost LDR Sensors for Solar Intensity Measurement. Proceedings of the 2024 IEEE International Instrumentation and Measurement Technology Conference (I2MTC).

[8] Yu B., Yin H., Zhu Z. (2018). Spatio-Temporal Graph Convolutional Networks: A Deep Learning Framework for Traffic Forecasting. Proceedings of the Twenty-Seventh International Joint Conference on Artificial Intelligence (IJCAI-18).

[9] Li Y., Yu R., Shahabi C., Liu Y. (2017). Diffusion Convolutional Recurrent Neural Network: Data-Driven Traffic Forecasting. arXiv.

[10] Wu Z., Pan S., Long G., Jiang J., Zhang C. (2019). Graph WaveNet for Deep Spatial-Temporal Graph Modeling. IJCAI’19: Proceedings of the 28th International Joint Conference on Artificial Intelligence.

[11] Longa A., Lachi V., Santin G., Bianchini M., Lepri B., Lio P., Scarselli F., Passerini A. (2023). Graph neural networks for temporal graphs: State of the art, open challenges, and opportunities. arXiv.

[12] Chabalala V., Rudolph C., Mosala K., Nkadimeng E.K., Mosomane C., Mathaha T., Basu P., Mahboob M.A., Kong J., Bragazzi N. (2026). Spatiotemporal Graph Neural Networks for PM_2.5_ Concentration Forecasting. Air.

[13] Pan Z., Xu L., Chen N. (2025). Combining graph neural network and convolutional LSTM network for multistep soil moisture spatiotemporal prediction. J. Hydrol..

[14] Tuo Y., Wirthensohn M., Disse M. Spatio-Temporal Graph Neural Networks for Soil Moisture Drought Forecasting: Adaptability, Predictability, and Interpretability. Proceedings of the AGU Fall Meeting 2023.

[15] Akkala A., Boubrahimi S.F., Hamdi S.M., Hosseinzadeh P., Nassar A. (2025). Spatio-Temporal Graph Neural Networks for Streamflow Prediction in the Upper Colorado Basin. Hydrology.

[16] Feng J., Sha H., Ding Y., Yan L., Yu Z. (2022). Graph convolution based spatial-temporal attention LSTM model for flood forecasting. Proceedings of the 2022 International Joint Conference on Neural Networks (IJCNN).

[17] Costanti F., Cappelli I., Ceroni E.G., Bianchini M., Fort A. (2025). Foliar Wetness Prediction Using Sensor Network Data and WaveNet-Based Deep Learning Models. Proceedings of the 2025 IEEE International Conference on Metrology for eXtended Reality, Artificial Intelligence and Neural Engineering (MetroXRAINE).

[18] He K., Chen X., Xie S., Li Y., Dollár P., Girshick R. Masked autoencoders are scalable vision learners. Proceedings of the IEEE/CVF Conference on Computer Vision and Pattern Recognition.

[19] Feichtenhofer C., Li Y., He K. (2022). Masked autoencoders as spatiotemporal learners. Adv. Neural Inf. Process. Syst..

[20] Jin G., Liang Y., Fang Y., Shao Z., Huang J., Zhang J., Zheng Y. (2023). Spatio-temporal graph neural networks for predictive learning in urban computing: A survey. IEEE Trans. Knowl. Data Eng..

[21] Zhang Q., Gao X., Wang H., Huang D., Yiu S.M., Yin H. HGAurban: Heterogeneous Graph Autoencoding for Urban Spatial-Temporal Learning. Proceedings of the 34th ACM International Conference on Information and Knowledge Management.

[22] Zhao F., Cao X., Zhao J., Duan Y., Yang X., Zhang X. (2025). Masked graph autoencoder-based multi-agent dynamic relational inference model for trajectory prediction. Neurocomputing.

[23] Costanti F., Cappelli I., Fort A., Ceroni E.G., Bianchini M. LSTM-based Siamese Networks for Fault Detection in Meteorological Time Series Data. Proceedings of the 2024 IEEE International Conference on Metrology for eXtended Reality, Artificial Intelligence and Neural Engineering (MetroXRAINE).

[24] Che Z., Purushotham S., Cho K., Sontag D., Liu Y. (2018). Recurrent neural networks for multivariate time series with missing values. Sci. Rep..

[25] Tang H., Yang H., Zhang W. (2025). DAHG: A Dynamic Augmented Heterogeneous Graph Framework for Precipitation Forecasting with Incomplete Data. Information.

[26] Sasal L., Busby D., Hadid A. (2024). Tempokgat: A novel graph attention network approach for temporal graph analysis. Proceedings of the International Conference on Neural Information Processing.

[27] Dimitri G.M., Cappelli I., Scarselli F., Fort A., Gori M. Graph Neural Networks for Missing Data Imputation in Time Series from Meteorological Sensors. Proceedings of the 2024 IEEE International Conference on Metrology for eXtended Reality, Artificial Intelligence and Neural Engineering (MetroXRAINE).

[28] Chung J., Gulcehre C., Cho K., Bengio Y. (2014). Empirical Evaluation of Gated Recurrent Neural Networks on Sequence Modeling. arXiv.

[29] Hamilton W., Ying Z., Leskovec J. (2017). Inductive representation learning on large graphs. Adv. Neural Inf. Process. Syst..

[30] Lence A., Granese F., Fall A., Hanczar B., Salem J.E., Zucker J.D., Prifti E. (2025). ECGrecover: A deep learning approach for electrocardiogram signal completion. Proceedings of the 31st ACM SIGKDD Conference on Knowledge Discovery and Data Mining V.1.

[31] Liang Y., Zhang Y., Chen F., Lu J., Lin Z. (2026). Decoding Speech Envelopes from Electroencephalogram with a Contrastive Pearson Correlation Coefficient Loss. arXiv.

[32] Lambert T., Gilman P., Lilienthal P. (2006). Micropower system modeling with HOMER. Integr. Altern. Sources Energy.

[33] Akiba T., Sano S., Yanase T., Ohta T., Koyama M. Optuna: A next-generation hyperparameter optimization framework. Proceedings of the 25th ACM SIGKDD International Conference on Knowledge Discovery & Data Mining.

